# TIMP-1 and CD82, a promising combined evaluation marker for PDAC

**DOI:** 10.18632/oncotarget.14133

**Published:** 2016-12-24

**Authors:** Jiexin Zhang, Tijun Wu, Shanshan Zhan, Nan Qiao, Xu Zhang, Yunxia Zhu, Nan Yang, Yujie Sun, Xin A Zhang, David Bleich, Xiao Han

**Affiliations:** ^1^ Department of Laboratory Medicine, The First Affiliated Hospital of Nanjing Medical University, Nanjing, China; ^2^ Key Laboratory of Human Functional Genomics of Jiangsu Province, Nanjing Medical University, Nanjing, China; ^3^ Stephenson Cancer Center and Department of Physiology, University of Oklahoma Health Science Center, Oklahoma, OK, USA; ^4^ Rutgers New Jersey Medical School, Newark, NJ, USA

**Keywords:** TIMP-1, CD82, interaction, cell motility, PDAC

## Abstract

Tissue inhibitor of metalloproteinases-1 (TIMP-1) is a widely secreted protein that regulates cell motility, proliferation, and apoptosis. Although it is recognized that TIMP-1–tetraspanin CD63 regulates epithelial cell apoptosis and proliferation, how TIMP-1 controls cell motility is not well understood. In this study, we identify tetraspanin CD82 (also called KAI1) as a component of the promiscuous TIMP-1 interacting protein complex on cell surface of human pancreatic adenocarcinoma cells. CD82 directly binds to TIMP-1 N-terminal region through its large extracellular loop and co-localizes with TIMP-1 in both cancer cell lines and clinical samples. Moreover, CD82 facilitates membrane-bound TIMP-1 endocytosis, which significantly contributes to the anti-migration effect of TIMP-1. CD82 silencing partially eliminates these functions. TIMP-1 and CD82 expression status in patients with pancreatic ductal adenocarcinoma (PDAC) might demonstrate future usefulness as a differentiation marker and give us new insight into tumorigenic metastatic potential.

## INTRODUCTION

Tissue inhibitors of metalloproteinases (TIMPs) are well known natural inhibitors of matrix metalloproteinases (MMPs); and their regulation of extracellular matrix remodeling is one of the most critical rate-limiting steps in the process of metastasis [1]. The non-covalent binding of TIMPs to MMPs, which are secreted by cells, reverses the unbalanced proteolytic activity of tumors and prevents local cell migration and invasion [2]. Of four known TIMPs (TIMP-1, -2, -3, -4), TIMP-1 is of particular interest because it promotes cell proliferation and inhibits apoptosis independent of its MMP-inhibitory activity [3, 4]. Previous studies have demonstrated that TIMP-1 binds a proMMP9/CD44 complex that facilitates erythroid cell survival and is localized to the cell surface [5]. However, other TIMP-1 complexes as yet not described are likely to participate in cellular signaling and trafficking given its pleotrophic functions.

We previously demonstrated that TIMP-1 dramatically inhibited cytokine-induced apoptosis in damage-sensitive rat pancreatic islets. It also partially restored glucose-stimulated insulin secretion [6]. Jung and colleagues showed that CD63 is a TIMP-1–interacting protein on cell surface of MCF10A breast epithelial cells that participated in TIMP-1–induced cell survival [7]. Here too, TIMP-1 mediated its cell signaling function through an MMP-independent mechanism [8, 9]. However, other potential proteins might interact with the TIMP-1/CD63 complex to mediate differential effects on cell growth, intracellular signaling and migration.

CD63 belongs to the tetraspanin family of proteins which were originally known as tetraspanin transmembrane antigens or transmembrane 4 superfamily proteins [10]. Tetraspanins, widely distributed in a wide range of tissue and cell types, including cancer cells [11], are defined by their four transmembrane domains and two differently sized extracellular loops Along with diverse partners such as integrins, immunoglobulin superfamily members, major histocompatibility complex molecules, and other transmembrane proteins [12], tetraspanins form tetraspanin-enriched microdomains (TEMs) on cell surface. These are now widely accepted as a new, specialized membrane region known as a tetraspanin web [13]. The tetraspanin web participates in several physiological/pathophysiological processes, including cell adhesion, motility, activation and cell–cell fusion [14]. So far, the tetraspanin family has at least 33 members, such as CD82, CD9 and CD81 [15, 16].

In this study, we describe our further analysis of the tetraspanin CD82. Particularly, we provide evidence that TIMP-1 directly binds to CD82. Additionally, the TIMP-1–CD82 complex interacts in an ordered manner in pancreatic ductal adenocarcinoma (PDAC).

## RESULTS

### Cell surface CD82 binds to TIMP-1

**Figure 1:**
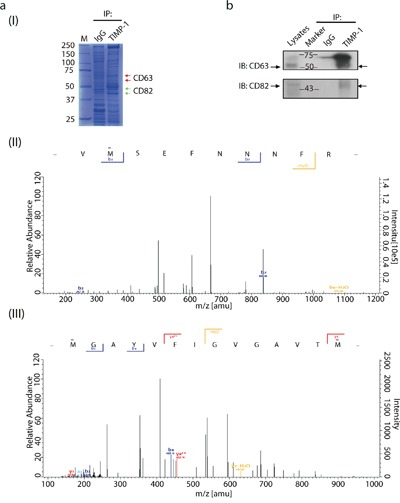
Protein-protein interaction exists between CD82 and TIMP-1 **a**. LC-MS/MS analysis of protein complex coimmunoprecipitated with TIMP-1 in PANC-1 cells. (I) Coomassie blue staining of 10% SDS-PAGE gel. Red arrowheads indicate protein complexes in which CD63 was detected. Green arrowheads indicate protein complexes in which CD82 was detected. (II) A digested peptide from CD63. (III) A digested peptide from CD82. **b**. Coimmunoprecipitation of endogenous TIMP-1 with CD63 or CD82 in PANC-1 cells. Cell lysates were immunoprecipitated with rabbit anti–TIMP-1 pAb or control IgG, followed by immunoblotting with mouse anti-CD63 mAb or mouse anti-CD82 mAb. Arrowheads indicate the immunoreactive bands of CD63 or CD82.

**Figure 2:**
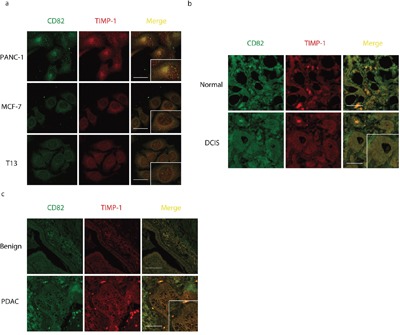
CD82 co-localizes with TIMP-1 in adenocarcinoma cells **a**. TIMP-1 co-localization with CD82 in cell lines, pearson's coefficient was 0.89±0.13 in PANC-1, 0.46±0.07 in MCF-7, 0.40±0.14 in T13. Scale bar = 20μm. **b**. *In vivo* TIMP-1 co-localization with CD82 in DCIS biopsies, pearson's coefficient was 0.26±0.01. Cells were double-immunostained with mouse anti-CD82 mAb and rabbit anti–TIMP-1 pAb. Superimposed areas are in yellow. Normal: healthy controls in the same DCIS biopsy sample. Scale bar = 200μm. **c**. *In vivo* TIMP-1 co-localization with CD82 in PDAC biopsies, pearson's coefficient was 0.46±0.18. Cells were double-immunostained with mouse anti-CD82 mAb and rabbit anti–TIMP-1 pAb. Benign: healthy/atypical hyperplasia control in the same PDAC biopsy sample. Scale bar = 200μm.

To test the possibility of TIMP-1 interacting with other tetraspanins, we used LC-MS/MS to analyze protein complexes co-immunoprecipitated with TIMP-1 [Figure 1a (I)]. As expected [8], we detected unique peptides of CD63 [Figure 1a (II)]. Interestingly, we also found CD82, another member belongs to tetraspanins family [Figure 1a (III)]. Both the molecular weight (data not shown) and membrane localization rendered CD82 a highly promising candidate. Given the high stringency of Triton X-100, That endogenous TIMP-1 co-immunoprecipitation with CD82 further encouraged TIMP-1–CD82 direct binding (Figure 1b). Next, TIMP-1 co-localization with CD82 was assessed by confocal microscopic analysis *in vitro*. Three human adenocarcinoma cell lines, PANC-1 (pancreatic), MCF-7 (breast), and T13 (TIMP-1–overexpressing MCF-7 clone) were cultured under basal conditions and stained for immunofluorescence analysis. All cell lines had a dotted staining pattern, particularly PANC-1 cells (Figure 2a, Pearson's coefficient was 0.89±0.13 in PANC-1, 0.46±0.07 in MCF-7, 0.40±0.14 in T13). Varied signal strength was due to the unequal TIMP-1 and CD82 expression in the cells: they were lowest in MCF-7 cells (data not shown). *In vivo*, biopsies from patients diagnosed with breast ductal carcinoma in situ (DCIS; Supplementary Figure 1a) and pancreatic ductal adenocarcinoma (PDAC; Supplementary Figure 1b) were immunostained with antibodies against TIMP-1 and CD82. Their co-localization was significantly enhanced both on surface of and inside the carcinoma cells, although both normal breast epithelial cells and DCIS demonstrated co-localization of TIMP-1 and CD82. (Figures 2b and Figure 2c, Pearson's coefficient was 0.26±0.01 in DCIS, 0.46±0.18 in PDAC). Negative controls, where only secondary antibodies were used, are shown in Supplementary Figure 1c. As demonstrated here, ductal structures were not visualized using only negative antibody controls so co-localized images were not obtained.

**Figure 3:**
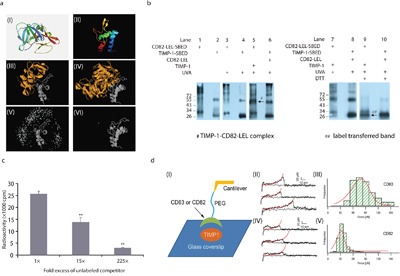
*In vitro* CD82 binds to TIMP-1 N-terminal through its LEL **a**. Bioinformatics analysis of TIMP-1 and CD82-LEL binding. (I) Ribbon representation of TIMP-1 (amino acids 24-204) based on the SWISS-MODEL template 1UEAB. The modeled homolog shares 98.9% similarity with TIMP-1 (amino acids 24-204). (II) CD82-LEL (amino acids 111-228) 3D structure by Phyre. It covers 64% of the LEL with 99.8% confidence, and presented the tetraspanin family characteristic of an α-helix structure before the conserved CCG motif.^16^ (III and IV) TIMP-1 and CD82-LEL (III) and TIMP-1–MMP3 and CD82-LEL (IV) top protein–protein docking results. Notably, the original TIMP-1–MMP3 data (PDB accession: 1UEA) contained two copies of the complex. We used Swiss-PdbViewer to simplify it into a single copy. (V and VI) Ligand center of mass positions for the top 500 ZDOCK models corresponding to III and IV, respectively. **b**. TIMP-1 and CD82-LEL binding by chemical cross-linking experiments. Lanes 1 and 2: CD82-LEL–SBED and TIMP-1–SBED in non-UVA condition, respectively. Lanes 3 and 4: CD82-LEL–SBED and TIMP-1–SBED in UVA condition, respectively. Lane 5: CD82-LEL–SBED mixed with recombinant TIMP-1 followed by UVA exposure. Lane 6: TIMP-1–SBED mixed with recombinant CD82-LEL followed by UVA exposure. Lanes 7 and 8: Same as lanes 5 and 6. Lanes 9 and 10: Same as lanes 7 and 8, but supplemented with DTT. Arrowheads indicate target bands. **c**. TIMP-1 and CD82-LEL binding by competitive binding analysis. Molar excess CD82-LEL competed with 125I-labeled CD82-LEL to bind to TIMP-1. Data are representative of two independent experiments performed in duplicate. Error bars represent *SD*. **p < 0.01. **d**. Single-molecule force spectroscopy characterization of the binding strength between TIMP-1 and CD63-LEL/CD82-LEL. (I) Experimental scheme. (II) Typical traces obtained from single-molecule force spectroscopy measurement for TIMP-1–CD63-LEL binding. (III) Distribution of the unbinding forces between TIMP-1 and CD63-LEL. (IV) Representative unbinding traces of TIMP-1–CD82-LEL. (V) Distribution of the unbinding forces between TIMP-1 and CD82-LEL.

Next, TIMP-1–CD82 binding pattern was analyzed using bioinformatics. The modeled homolog in Figure 3a (I) shared 98.9% similarity with TIMP-1 (amino acids 24-204). Although the small extracellular loop of CD82 may not be long enough to interact with certain proteins, it maintained the large extracellular loop (LEL) spatial conformation [17]. Since the only resolved crystal structure of the tetraspanin family is CD81 LEL, we used the Phyre server to predicte the structure of CD82-LEL (amino acids 111-228). As shown in Figure 3a (II), the output covered 64% of the LEL with 99.8% confidence, and presented the tetraspanin family characteristic of an α-helix structure before the conserved CCG motif [18]. Before uploading information for final analysis, we included the potential binding sites in CD82-LEL: ASN129, CYS149-151GLY, ASN157, CYS174, CYS176, and CYS216. Figure 3a (III) depicted the top docking result of TIMP-1 with CD82-LEL. Then, we examined the possibility of TIMP-1 binding to CD82-LEL in the presence of MMPs. The prototype selected was the first resolved TIMP-1–MMP3 crystal structure of all known TIMP-1 complexes [19]. Notably, the original TIMP-1–MMP3 data (PDB accession: 1UEA) contained two copies of the complex. Therefore, we used Swiss-PdbViewer to simplify it into a single copy. Before uploading for ZDOCK analysis, MMP binding sites in TIMP-1 (CYS24-29PRO, VAL52, THR56-58TYR, ALA88-93CYS, THR120-123SER, and LEU156-157SER) were blocked [20]. Figure 3a (IV) shows that TIMP-1 bridged MMP3 to CD82-LEL through non–MMP-binding sites. However, MMP3–TIMP-1–CD82-LEL binding probability [Figure 3a (V)] decreased dramatically compared with TIMP-1–CD82-LEL [Figure 3a (VI)], which indicated that the interaction between TIMP-1 and CD82-LEL could be weakened under the condition where MMP3 had already combined to TIMP-1. To verify these predictions above, we performed protein chemical cross-linking experiments between recombinant TIMP-1 and CD82-LEL *in vitro*. Sulfo-SBED used in the cross-linking experiments is a heterobifunctional chemical crosslinker capable of covalently attaching to primary amines at N-terminus [Supplementary Figure 2a (I)]. Unlike typical crosslinkers, Sulfo-SBED also includes a biotin group and a cleavable disulfide spacer arm. When SBED-labeled protein is exposed to UV light, an interacting protein will be captured by the photoreactive aryl azide moiety. Upon reduction of the disulfide bond, the biotin label will be transferred to the interacting protein. The biotinylated interacting protein can be detected by western blotting using streptavidin-HRP [Supplementary Figure 2a (II)]. Then, three groups (Figure 3b, lanes 1-6) were established in parallel. N-terminal biotinylated CD82-LEL (CD82-LEL-SBED) bands ranged 26-250 kDa (lanes 1 and 3) due to glycosylation [10] and/or homodimerization, trimerization, and tetramerization [21]. N-terminal biotinylated TIMP-1 (TIMP-1-SBED) had two clear monomer (~20 kDa) and homodimer (~55 kDa) bands (lanes 2 and 4). Similar bands were detected when no-biotinylated recombinant protein CD82-LEL or TIMP-1 were cross-added to TIMP-1-SBED or CD82-LEL-SBED respectively, followed by ultraviolet A (UVA) exposure (lane 5 vs. lanes 1 and 3; lane 6 vs. lanes 2 and 4), except for a ~50 kDa band corresponding to the molecular weight of the TIMP-1–CD82-LEL complex (lane 5, as arrow pointed to). DTT was then applied to reduce disulfide bonds in the cross-linking reagent to transfer the biotin label from the biotinylated protein to the interacting protein (lanes 7-10). Following disulfide bond reduction, the prominent band (~50 kDa) which represented CD82-LEL-SBED dramatically shifted to a lower molecular weight (~20 kDa) (lane 7 vs. 9, as arrow pointed to), indicating that the biotin label had transferred from CD82-LEL to TIMP-1 (~20 kDa). This meant that CD82-LEL could bind to TIMP-1 *in vitro*. However, no label transfer was detected in lane 10, which revealed that TIMP-1 could not bind to CD82-LEL if its N-terminus was blocked with crosslinker. Western blotting of TIMP-1-SBED and CD82-LEL-SBED treated with DTT or not were shown in Supplementary Figure 2b (I). In addition, we repeated the cross-linking experiments between recombinant protein CD82-LEL and TIMP-2 which is a high homology to TIMP-1 [22]. However, the band of CD82-LEL-SBED did not transfer to TIMP-2 under these conditions [Supplementary Figure 2b (II)]. Therefore, the binding between CD82-LEL and TIMP-1 was specific.

Competitive binding analysis further validated the affinity and specificity of TIMP-1 and CD82-LEL binding (Figure 3c). The presence of increasing amounts of CD82-LEL caused a significant and dose-dependent decrease in 125I–CD82-LEL binding to TIMP-1. Single-molecule force spectroscopy characterization of the binding strength between TIMP-1 and CD82-LEL was accurately performed [Figure 3d (I)]. Figure 3d (II) depicted the typical traces of the positive control TIMP-1–CD63-LEL binding. The average unbinding force between TIMP-1 and CD63-LEL was ~80 pN [Figure 3d (III)], whereas the unbinding force between TIMP-1 and CD82-LEL was centered at ~35 pN [Figure 3d (IV) and (V)]. All of the above exposed, TIMP-1 directly binds to CD82 through its N-terminal *in vitro*. Co-localization of TIMP-1 and CD82 observed in breast ductal carcinoma and pancreatic ductal adenocarcinoma may arise from this.

### CD82 plays a role in TIMP-1 cytoplasmic translocation in PANC-1 and MCF-7 cells

**Figure 4:**
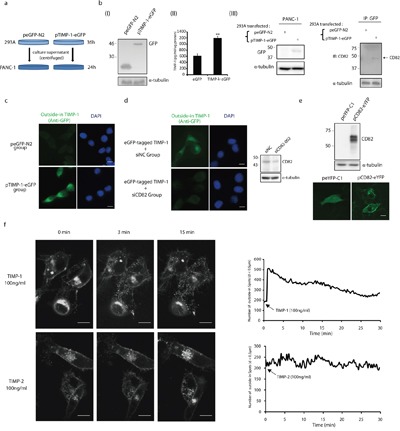
CD82 participates in TIMP-1 cytoplasmic translocation in PANC-1 cells **a**. Culture system model of 293A cells and PANC-1 cells. **b**. The culture system was effective. (I) 293A cells were transfected with peGFP-N2 or pTIMP-1–eGFP. Cell lysates were immunoblotted with anti-GFP and anti–α-tubulin mAbs. (II) ELISA of TIMP-1 in 293A culture supernatant. TIMP-1 level in culture supernatant from 293A cells transfected with pTIMP-1-eGFP was almost twice as much as it from 293A cells transfected with eGFP. (III) PANC-1 cells were treated with culture supernatant from eGFP/eGFP-TIMP-1 transfected 293A cells. Outside-in GFP was detectable in PANC-1 cells. Cell lysates were immunoblotted with anti-GFP and anti–α-tubulin mAbs. Endogenous CD82 could be immunoprecipitated by outside-in TIMP-1 (eGFP-tagged) in PANC-1 (as arrowhead pointed at). **c**. TIMP-1–eGFP membrane–cytoplasm translocation in PANC-1 cells. PANC-1 cells were cultured with culture supernatant of 293A cells transfected with peGFP-N2 or pTIMP-1–eGFP. PANC-1 cells were immunostained with mouse anti-GFP mAb and counterstained with DAPI. **d**. CD82 mediated TIMP-1–eGFP membrane–cytoplasm translocation in PANC-1 cells. PANC-1 cells which were transfected with siNC (100 nM) or siCD82 (100 nM) for 48h were treated with culture supernatant of 293A cells transfected with peGFP-N2 or pTIMP-1–eGFP. PANC-1 cells were immunostained with mouse anti-GFP mAb and counterstained with DAPI. Scale bar = 20μm. CD82 deletion was confirmed by western blotting. **e**. CD82-eYFP fusion protein overexpression by pCD82-eYFP transfection. 293A cells were transfected with peYFP-N2 or pCD82-eYFP for 24 h. Cell lysates (using 1% Triton X-100) were immunoblotted with mouse anti-CD82 and anti–α-tubulin mAbs. Images shown are representative of eYFP and CD82-eYFP distribution in live PANC-1 cells. Scale bar = 20μm. **f**. TIMP-1 protein but not TIMP-2 triggers CD82 leaving from cytomembrane into cytoplasm. Images shown are representative of intense foci of CD82-eYFP fluorescence in the 0^th^, 3^th^, 15^th^ minute since TIMP-1 or TIMP-2 protein (100 ng/ml) addition in live PANC-1 cells. The scatter diagram quantified the images in the right by calculating outside-in spots (less than 0.5μm, reflecting CD82) in PANC-1 cytoplasm using Imaris8.0.

*In vivo*, TIMP-1 is ubiquitously synthesized and released into the extracellular microenvironment. To mimic the cross-talk between TIMP-1 and adenocarcinoma cells, a culture system was established as shown in Figure 4a. Firstly, 293A cells were transfected with peGFP-N2 (control) or pTIMP-1-eGFP for almost 36h. TIMP-1–eGFP expression was examined in 293A cells [Figure 4b (I)]. Culture supernatant was collected and centrifuged respectively, then used as culture media for PANC-1 cells for 24h. ELISA of TIMP-1 in 293A culture supernatant [Figure 4b (II)] and western blotting [Figure 4b (III)] to detect outside-in eGFP in PANC-1 cells were done to ensure that this kind of culture method was effective. Additionally, endogenous CD82 could coimmunoprecipitation with outside-in TIMP-1 (eGFP-tagged) in PANC-1 [Figure 4b (III), as arrow pointed to]. After culture, green fluorescence was detected inside PANC-1 cells cultured with supernatant from 293A cells transfected with TIMP-1–eGFP, but it was barely detectable in the eGFP group (Figure 4c). RNA interference was used to assess the pivotal role of CD82 in extracellular TIMP-1 localization in PANC-1 (Figure 4d). Culture media of PANC-1 which had been transfected with siNC or siCD82 were replaced with culture supernatant which containing eGFP-tagged TIMP-1(mentioned above). After 24 hours, it was obvious that green fluorescence in the eGFP-tagged TIMP-1+siCD82 group had mostly vanished compared to that in the eGFP-tagged TIMP-1+siNC group (Figure 4d) because eGFP-tagged TIMP-1 could hardly be transferred into cytoplasm without CD82. According to an earlier study [23], CD82 can be internalized, indicating that it is a component of a mobile protein complex on surface of/inside cells. To examine whether the sub-cellular localization of TIMP-1 concurred with CD82 during interaction, we used live cell imaging to study the localization of CD82-eYFP fusion protein before and after triggering by TIMP-1 protein. In transiently transfected PANC-1 cells, CD82-eYFP fusion proteins were distributed mainly on the plasma membrane in most cells, which was consistent with that of previous findings (Figure 4e) [24]. As soon as addition of TIMP-1 recombinant protein, large amounts of intense foci of CD82-eYFP fluorescence were immediately detected in the cytoplasm and peaked in 3 minutes (Figure 4f, upper; Supplementary Movie 1), then was sustained intracellularly. However, no outside to inside transfer of green fluorescent dots were noted with TIMP-2 under similar experimental conditions (Figure 4f, lower). Interestingly, we also observed that filopodia present on the surface of PANC-1 cells underwent retraction within 15 min after TIMP-1 was applied which coincided with accumulating green dots intracellularly (Supplementary Movie 1). These results suggest that TIMP-1–CD82 complex translocated into PANC-1 cell cytoplasm after TIMP-1 binding-activation.

TIMP-1 and CD82 co-localization on breast epithelial cell–derived cell membranes prompted us to investigate further. A culture system similar to that of PANC-1 cells was established (Supplementary Figure 3a). eGFP-tagged TIMP-1 was also present in the cytoplasm of MCF-7 cells as it happened in PANC-1 cells. Endogenous membrane CD82 depletion by siRNA significantly hindered TIMP-1–eGFP translocation (Supplementary Figure 3c). These data indicated that TIMP-1 endocytosis in MCF-7 cells is similar to that of PANC-1 cells.

### CD82 ensures TIMP-1 inhibition of PANC-1 cell migration

**Figure 5:**
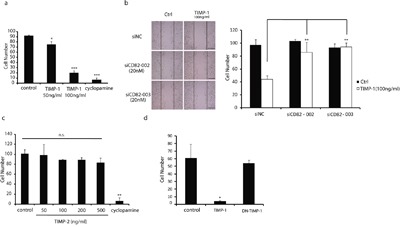
CD82 ensures TIMP-1 inhibition of PANC-1 cell migration **a**. Migration inhibitory effect of cyclopamine and TIMP-1 in PANC-1 cells. Cells were treated with cyclopamine (10 μM) or TIMP-1 (50, 100 ng/mL) for 24 h. **b**. Mild CD82 deletion weakened TIMP-1 inhibition of PANC-1 cell migration. Cells were transfected with siNC (20 nM) or two independent siRNAs (20 nM) targeting CD82 for 48h. Cells were then treated with TIMP-1 (100 ng/mL) for 6 h. Scale bar = 60μm. **c**. TIMP-2 had no inhibitory effect on PANC-1 cell migration. Cells were treated with TIMP-2 or cyclopamine (10 μM) for 24 h. **d**. DN–TIMP-1 significantly weakened TIMP-1 inhibition of PANC-1 cell migration. Cells were treated with TIMP-1 (100 ng/mL) or DN–TIMP-1 (100 ng/mL) for 18 h. Error bars represent *SD*. *p < 0.05; **p<0.01; ***p<0.001; ns denotes no statistical significance.

The role of TIMP-1 in pancreatic cancer has not received much attention to date. Previous studies found that TIMP-1–expressing pancreatic cancer cells were significantly less invasive, and mice receiving TIMP-1 adenovirus showed reduced cancer cell growth and prolonged survival rates [25]. To investigate that whether TIMP-1 could reduce pancreatic cancer cell migration, exogenous recombinant TIMP-1 was added to the culture medium of poorly differentiated PANC-1 cells at common concentrations of 50, 100 ng/mL [6, 26], followed by a wound closure assay. Cyclopamine (10 μM) was used as a positive control which is widely known as a Hedgehog signaling pathway inhibitor and being able to suppress cancer metastasis, including pancreatic cancer metastasis [27]. As shown in Figure 5a, TIMP-1 effectively inhibited PANC-1 cell migration, although not as strongly as cyclopamine, with a maximum effect at 100 ng/mL. Next, a set of wound closure assays was redesigned following a timetable of 6h to investigate the effects of interaction between CD82 and TIMP-1 on PANC-1 cell migration. CD82 is recognized as a metastasis suppressor in multiple carcinomas [27, 28]. In order to avoid that CD82 deletion alone could weaken cell migration irrespective of TIMP-1 status, the concentration of siCD82 in this wound closure assay was lowered to 20 nM at which molality PANC-1 cell motility was not be obviously induced (Figure 5b, left). Surprisingly, the inhibitory effect of TIMP-1 on cell migration could still be rescued in the case that CD82 expression was decreased slightly (Figure 5b, right). Thus, we believe that TIMP-1 plays a role in the suppression of cancer cell migration by interacting with CD82.

To evaluate the specificity of TIMP-1 inhibition of migration, TIMP-2 was selected for its high homology to TIMP-1 and analogous anti-cancer effect [28]. Figure 5c shows that recombinant TIMP-2 failed to inhibit PANC-1 cell migration. TIMP-1 N-terminal was seemed vital to its binding to CD82 (Figure 3b, lanes 8 vs. 10). To mimic the interaction between TIMP-1 and MMPs *in vivo*, i.e., the active N-terminal of TIMP-1 is occupied by MMPs, we used the Sulfo-SBED cross-linking reagent again. It can form a tight amide bond with the first amino acid of TIMP-1 at the N-terminal to disable TIMP-1 [dominant negative TIMP-1 (DN–TIMP-1)]. As is shown in Figure 5d, the migration inhibitory effect of DN–TIMP-1 was significantly weakened.

To exclude the influence of TIMP-1 on the cell cycle, siCD82 and parental PANC-1 cells were subjected to flow cytometry after TIMP-1 stimulation (Supplementary Figure 4a). Here we demonstrate that TIMP-1 specifically inhibited PANC-1 cell migration without affecting cell growth.

**Figure 6:**
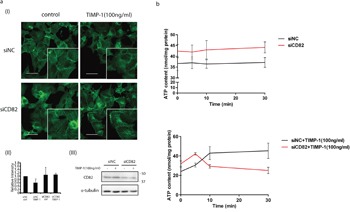
Mechanisms of that TIMP-1 exclusively inhibited PANC-1 cell migration through CD82 **a**. TIMP-1 modified cytoskeleton remodeling via CD82. Cells were transfected with siNC or siCD82 and treated with TIMP-1 (100 ng/mL) for 18 h. (I) Cells were stained with fluorescent phalloidin, counterstained with DAPI and analyzed. Scale bar = 20μm. (II) Relative intensity of F-actins was qualified by ImageJ. (III) CD82 deletion was confirmed by western blotting. **b**. TIMP-1 increased intercellular ATP concentration through CD82. Cells were transfected with siNC or siCD82 and went through serum starvation for 6 h. Cell were lysed for ATP detection without treatment or after TIMP-1 (100 ng/mL) stimulation. Error bars represent *SD*. *p < 0.05; **p<0.01; ***p<0.001.

Integrins localize in most TEMs. The tetraspanin–integrin complex at the leading edge of the cell membranes modifies cytoskeleton remodeling [29]. Considering that TIMP-1 addition induced filopodia quick retraction (Supplementary Movie 1) and filopodia contains crosslinked actin filaments which attached to cell membrane through integrins [30], fluorescent phalloidin staining was performed to investigate the distribution pattern of F-actin in PANC-1 cells. F-actins in the TIMP-1 treated/CD82 wild-type group were loose and diffuse [Figure 6a (I), upper right] compared with control/CD82 wild-type group [Figure 6a (I), upper left]. Following CD82 depletion, linear actins were observed [Figure 6a (I)] in both of siNC and siCD82 groups regardless of TIMP-1 addition. In some cells, typical membrane filopodia were noted. In living cells, the free concentration of ATP is about tenfold higher than that of ADP, so most soluble actin subunits are in the “ATP form”. However, when the subunits are incorporated into filaments, they catalyze ATP. ATP concentration in siNC and siCD82 group did not change during 30 minutes (Figure 6b, upper). After TIMP-1 stimulation, ATP concentration of wild type PANC-1 cells accumulated gradually from 5 to 30 min, while that of siCD82 cells clearly dropped indicating cell movement (Figure 6b, lower).

Collectively, TIMP-1–CD82 axis exclusively inhibits PANC-1 cell migration by modifying actin cytoskeleton via an ATP-responsive pathway, and probably in an MMP-dependent manner.

### TIMP-1 and CD82 expression in PDAC patients

**Figure 7:**
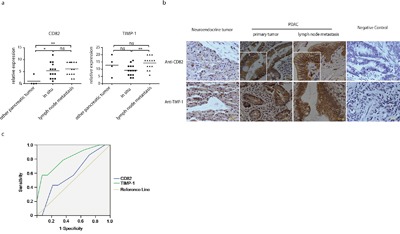
TIMP-1 and CD82 expression in PDAC patients **a**. Immunohistochemistry staining scores of TIMP-1 and CD82 in patients with pancreatic diseases (n=32). **b**. Cellular location of TIMP-1 and CD82. Photomicrographs represent TIMP-1 (rabbit anti–TIMP-1 pAb) and CD82 (mouse anti-CD82 mAb) immunostaining. Arrowheads delineate duct epithelial cells. Scale bar = 200μm. **c**. ROC curve was plotted to determine the accuracy of TIMP-1 as diagnostic test to discriminiate between PDAC with metastasis lesions and without metastasis lesions. *p < 0.05; **p<0.01; ns denotes no statistical significance.

We evaluated TIMP-1 and CD82 expression by immunohistochemistry in 32 cases of histological or cytological confirmed pancreatic diseases (Table 1). 28 cases (87.5%) were diagnosed with PDAC, and the others were 1 case of mucinous cystic tumor, 1 case of neuroendocrine tumor, 1 case of serous capsule adenoma, and 1 case of mucinous adenocarcinoma. Among PDAC patients, 22 cases (68.7%) were at low differentiation grade, and 6 cases (18.8%) were at high differentiation grade. PDAC patients with or without lymph node metastasis were 14 cases (43.7%) and 18 cases (56.3%) respectively. For staining evaluation, we used a semi-quantitative scoring criterion: positive cell percentage (score 0~5) × staining intensity (score 0~5). As shown in Figure 7a, CD82 expression was only activated in PDAC and the mucinous cystic tumor case, and it did not undergo further increase when lesions progressed. On the contrary, TIMP-1 expression level was high in all three groups. Of notice, CD82 staining was restricted to plasma membrane in patients with lymph node metastasis, but was distributed diffusely in the cytoplasm as well as on the plasma membrane in primary tumor (Figure 7b). Further operating characteristic curve (ROC) analysis of TIMP-1 showed that its staining scores could discriminate between primary tumor and lymph node metastasis (area under the curve, 0.809; 95% CI, 0.649 to 0.969; Figure 7c). These data indicate that CD82 expression discriminates PDAC from most of other pancreatic tumors, and combined detection of TIMP-1 and CD82 helps to assess PDAC pathological stage.

**Table 1:** Patient demographics and baseline characteristics

	Parafin section (n=32)
Age (years)	65 (25–81)
Sex	
Men	18 (56.2%)
Women	14 (43.8%)
Histology	
adenocarcinoma	28 (87.5%)
other	4 (12.5%)
Differentiation grade	
Low (II–III)	22 (68.7%)
High (I–II/II)	6 (18.8%)
Unknown	4 (12.5%)
Lymph node metastasis	
Yes	14 (43.7%)
No	18 (56.3%)

Data are median (range) or number (%).

## DISCUSSION

**Figure 8:**
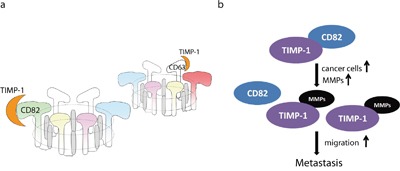
Models **a**. Theoretical model of the TIMP-1 receptor on the cell surface of pancreatic carcinoma cells. After directly binding to TIMP-1, CD82 or CD63 forms a hexameric complex containing five other tetraspanins. Each hexamer comprises a ring of six inner (SELs) and six outer parts (LELs). **b**. Hypothetical development of PDAC.

In the present study, we identified CD82 as a TIMP-1–binding cell surface protein according to four aspects: affinity, reversibility, specificity, and saturability. Palmitoylated CD82 specifically recruits interaction partners, including epidermal growth factor receptor (EGFR), EWI-2, integrin α6, c-Met and Vangl1, into signaling platforms such as lipid rafts and glycosynapses [31–33]. CD82 scaffolding assembles the other five family members, which are also in tetraspanin/partner-specific pairs, to form a tetraspanin–core complex on cell surface. Therefore, we believe that, at least in PDAC cells, TIMP-1 and its membrane-interacting tetraspanins (CD63 and CD82) do not conduct the traditional ligand–receptor interaction since the tetraspanins have no intrinsic enzymatic activity. We term this advanced form “ligand–TEMs” (Figure 8a). An earlier study showed that cholesterol depletion or ganglioside reduction eliminated CD82-containing complex formation and abrogated its inhibitory effect [34]. That study not only provides a credible solution to a long-standing debate about the relationship among TEMs, lipid rafts and glycol-synapses, but also indicates that mutiple interacting proteins and 3-D conformational interactions provide flexibility and functional heterogeneity of the TEMs.

In the species or tissue-derived cellular microenvironment (for example, PDAC in this study and lung cancer), such protein interactions help to explain the diversity and occasional divergent biology of TIMP-1.

Due to a tyrosine-based internalization motif at the C-terminal cytoplasmic domain, CD82 facilitates the removal of EGFR, integrin α6, and Vangl1 from the cell membrane [35]. It is widely accepted that its presence in the early endosome, lysosome and exosome [36] and its involvement in leukocyte antigen presentation [37] and the HIV infection process [38]. We observed CD82-eYFP fusion proteins endocytosis after TIMP-1 protein stimulation, suggesting the dynamic behavior of tetraspanin microdomains is also true in this study. The established cell culture system combined with immunofluorescence staining we used has three advantages: 1) the TIMP-1–eGFP fusion proteins are transported, processed and secreted in the same manner as endogenous TIMP-1, and it is traceable; 2) other proteins binding to TIMP-1 occur in parallel with MMP binding; 3) it mimics the extracellular environment. Our studies demonstrate that TIMP-1–eGFP selectively accumulates in the cytoplasm of PANC-1 and MCF-7 cells and silencing endogenous CD82 expression prominently eliminates TIMP-1 membrane–cytoplasm translocation. We assumed that extracellular TIMP-1 binds to CD82 on cell surface and is recruited into a function-related TEM, followed by endocytosis with CD82. This is the first study on the CD82 involvement in ligand endocytosis, illustrating its role in TIMP-1 intercellular trafficking and turnover. However, different staining patterns emerged in Figure 2a: co-localization of TIMP-1 and CD82 punctate in PANC-1, and perinuclear in MCF-7. We speculate that once transferred into cytoplasm TIMP-1 protein was degraded by lysosomes in PANC-1 cell, while in MCF-7 cells TIMP-1 protein may be processed through an alternative mechanism. Thus, in PANC-1 cells, co-localization of TIMP-1 and CD82 appeared mainly in the form of clustered dots in immunofluorescence, while in MCF-7 cells the co-localization seemed to disperse in the cytoplasm. In order to confirm our hypothesis, we used Lyso-Tracker Red, a red fluorescence probe for live cells, to label lysosomes in PANC-1 and MCF-7 which were treated with culture supernatant containing eGFP-tagged TIMP-1. It was obvious that outside-in TIMP-1 (green) merged with lysosomes (red) in PANC-1 much more frequently than it in MCF-7(Supplementary Figure 3d, Pearson's coefficient was 0.79±0.17 in PANC-1, 0.13±0.09 in MCF-7), which supported our hypothesis to some extent but still needs further investigation. The ambient dose of TIMP-1 might also be relevant to this observation. For example, if internalized TIMP-1 is at higher concentration than endogenous TIMP-1, the TIMP-1 staining pattern looks like an endosomal/lysosomal pattern as what we see in PANC-1 cells. If internalized TIMP-1 is much less concentrated than endogenous TIMP-1, then the TIMP-1 staining pattern looks like perinuclear pattern as what we see in MCF-7 cells. We will focus on this in the future study.

We also found that TIMP-1–CD82 synergistic inhibition of migration was already significant in the first 3 minutes after application of reagents (Figure 4f). So, TIMP-1 endocytosis probably led to desensitization of the TIMP-1 “receptor” or attenuation of TIMP-1–induced signaling. TIMP-1–CD82 interaction in PDAC is closely related to the particular function of CD82 that can cluster specifically at the edge of filopodial protrusions and regulate immediate cytoskeleton reorganization, whereas TIMP-1–CD63 complex in mammary gland is known to regulate cell apoptosis and proliferation. Moreover, the weaker binding strength of CD82 compared to CD63 also implies that CD82 targets different organelles and might be concentration dependent (Figure 3d). Therefore, more attention should be paid to mature TIMP-1 in the cytoplasm [39, 40].

Another novel piece of information provided by this study is the finding that TIMP-1 binding to CD82 requires its N-terminal domain to be available (Figure 3 and Figure 5d). Several clinical studies have shown that CD82 is related to metastases and is downregulated in the advanced stages of various epithelial malignancies, including prostate, colon, lung, pancreatic, breast, and ovarian cancers [41]. However, the mechanisms underlying CD82 regulation remain unknown. Gene mutation [42], loss of heterozygosity [43], and promoter mutation and hypermethylation [44] have been ruled out, whereas the possibilities of transcriptional and post-translational modification remain [45]. According to immunohistochemistry results of 32 patients in this study, we believe that defects in the N-terminus of TIMP-1 and CD82 endocytosis deficiency are both responsible for cancer development. Therefore, we propose a model of the natural history of PDAC (Figure 8b). At the beginning of carcinogenesis, TIMP-1 in the extracellular microenvironment binds to CD82 through its N-terminal. The anti-migratory effect coexists with the MMP-independent anti-apoptotic effect. As tumor progresses, cells loaded with MMPs accumulate, leading to TIMP-1 and MMP imbalance. More MMPs bind to the TIMP-1 N-termini. When TIMP-1–MMP complex can no longer interact with CD82 or CD82 fails to undergo endocytosis thereby impairing anti-migratory effects and inhibiting anti-apoptotic effects. These defects might predispose cells to take on a metastatic phenotype.

In pancreatic diseases, CD82 detection helps to discriminate PDAC from other tumors (96.9% in accordance with pathology diagnosis). CD82 membrane distribution pattern plus TIMP-1 staining score further confirmed PDAC metastasis status (100% in accordance with pathology diagnosis) when the overall survival rate is strikingly low. Drugs with analogous TIMP-1 N-terminal structure may be a promising therapeutic strategy in the future. Moreover, CD82 endocytosis recovery is needed to guarantee TIMP-1 anti-metastatic effect.

TIMP-1 is the first identified member of the vertebrate TIMP family and it appears to have the lowest rate of evolutionary change. Full understanding of TIMP-1 may reflect its hitherto unknown family characteristics. Our findings shed light on a new ligand–microdomain transmembrane signal transduction platform on cell surface, and highlight dynamic cross-talk between extracellular fluid and cancer cells mediated by TIMP-1 and CD82. We believe that in the future subclinical PDAC patients will benefit from combined TIMP-1 and CD82 pathological detection.

## MATERIALS AND METHODS

### Cells

The human pancreatic carcinoma cell line PANC-1 was obtained from ATCC. MCF-7 was a gift from Dr. Krontiris’ Laboratory at City of Hope National Medical Center in Los Angeles, USA. T13 is a TIMP-1 overexpression MCF-7 clone. 293A was purchased from Qbiogene, Inc., USA. We verified all cell identities. PANC-1 and 293A were grown in DMEM plus 10% FBS. MCF-7 and T13 were cultured in DMEM containing 2.5% FBS, 7.5% CBS. All culture medium were supplied with 100 U/ml penicillin and 100 U/ml streptomycin. The cultures were incubated at 37°C in a humidified atmosphere with 5% CO2. Cells were passaged every 2–3 days to obtain exponential growth.

### Reagents

Sulfo-SBED biotin label transfer reagent, Phosphate Buffered Saline (PBS) Pack, 1,4-Dithiothreitol (DTT), non-reducing sample buffer and Western Blot Stripping Buffer were purchased from Thermo Scientific (Rockford, USA). Recombinant human TIMP-1 (Cys 24-Ala 207), TIMP-2 (Cys 27-Pro 220) and CD82-LEL (Gly 111-Leu 228) were procured from Sino Biological, Inc. (Beijing, China). We purchased 125I-CD82-LEL from Beijing North Institute of Biological Technology (Beijing, China). We obtained CBS from PAA laboratories (Pittsburgh, USA) and FBS from Hyclone (Rockford, USA). RPMI-1640, DMEM, penicillin, and streptomycin were purchased from Gibco (Grand Island, USA). We procured First Strand cDNA Synthesis Kit, PrimeSTAR HS DNA Polymerase, and SYBR Green Real-time PCR Master Mix from TOYOBO (Osaka, Japan). Protease Inhibitor Cocktail came from Novagen (Darmstadt, Germany). Cyclopamine was obtained from BIOMOL International Lp (Shanghai, China). TRIzol Reagent and Lipofectamine 2000 were bought from Invitrogen (Grand Island, USA). ATP detection kit was bought from Beyotime (Shanghai, China). Immunofluorescence mounting medium and protein A/G-agarose beads were procured from Santa Cruz Biotechnology (Dallas, USA). Diaminobenzidine tetrahydrochloride (DAB) was purchased from Bioworld (Shanghai, China).

### Antibodies

Rabbit anti–TIMP-1 polyclonal antibody (pAb) was purchased from Abcam (Cambridge, USA). Mouse anti–CD82 monoclonal antibody (mAb), anti–GFP mAb, anti–α-tubulin mAb, and anti–β-Actin mAb were purchased from Santa Cruz Biotechnology. Stabilized Streptavidin-HRP Conjugate was obtained from Thermo Scientific. We purchased FITC-labeled phalloidin from Sigma (St. Louis, USA). We obtained horseradish peroxidase-conjugated secondary antibodies from Amersham Pharmacia Biotech (Piscataway, USA). Fluorescent-labeled secondary antibodies were purchased from Invitrogen.

### Coimmunoprecipitation

Two 10 cm dishes containing PANC-1 cells were incubated at 12-14°C for 5 h. Each membrane fraction (~4 mg/mL total protein) was solubilized for 30 minutes in immunoprecipitation (IP) buffer consisting of 50 mM Tris-HCl [pH 8.0], 150 mM NaCl, 1% Triton X-100, and 1% protein inhibitor cocktail on ice. Samples were centrifuged at 12 000 rpm for 20 minutes at 4°C to collect the supernatant. One part was lysate, and the other part was incubated with 40 μL pre-washed protein A/G-agarose beads for 1 h at 4°C with agitation. The same amount of rabbit anti–TIMP-1 pAb or control immunoglobulin G (IgG) was supplemented separately and agitated at 4°C overnight. Bound proteins were washed several times with IP buffer. Following sodium dodecyl sulfate–polyacrylamide gel electrophoresis (SDS-PAGE), samples were subjected to liquid chromatography–tandem mass spectrometry (LC-MS/MS; LTQ Exactive Orbitrap, Thermo Scientific, USA) or immunoblotted with mouse anti-CD82 mAb or mouse anti-CD63 mAb.

### Immunofluorescence staining

Cells were cultured on sterile cover slips, fixed in methanol/acetone 1:1 for 10 minutes and rinsed in PBS without or with permeabilization by 0.2 (w/v) Triton X-100 for 8 minutes. Non-specific binding was blocked with 5% normal goat serum in PBS for 1 hour at room temperature followed by primary antibodies (TIMP-1, dilution 1:100; CD82, dilution 1:100; GFP, 1:100; FITC-labeled phalloidin: 5 μg/ml) overnight at 4°C. The cover slips were washed in PBS three times for 5 minutes each time. Cells were incubated in fluorescent-labeled secondary antibodies (Alexa Fluor goat anti-mouse, goat anti-rabbit) at room temperature for 2 hours, followed by three washes with PBS. Permeabilized cells were counterstained with 4’, 6-diamidino-2-phenylindole (DAPI) for 5 minutes to stain the nucleus. Cover slips were mounted with antifade medium. Cells were visualized by a laser scanning microscope (LSM 710, Zeiss, USA) or a fluorescence microscope (Axio scope A1, Zeiss, USA) and photographs were captured using ZEN2009 or Axio vision.4.5, respectively. Co-localization were quantified with ImageJ.

### Patients and histological studies

We recruited 38 hospitalized patients. Eligible patients were those aged 25 or older with histologically or cytologically confirmed pancreatic diseases, breast ductal carcinoma, or colorectal carcinoma. Lymph node involvement was required for pathological metastasis confirmation. The study was approved by Ethics Institutional Review Board of the First Affiliated Hospital of Nanjing Medical University. All patients provided written informed consent before participation. Paraffin-embedded tissue sections (4 μm) were placed on glass slides. For histological study, sections (in duplicate for each individual) were baked at 60°C for 1 hour, deparaffinized in xylene for 30 minutes, and hydrated in a graded series of ethanol. One section was HE stained for histological examination; the other section was immersed in citrate buffer (pH 6.0) for antigen retrieval. A blocking solution (5% normal goat serum) was used to reduce non-specific antibody binding. Sections were subjected to immunofluorescence staining or immunohistochemistry.

### Bioinformatics analysis

SWISS-MODEL (version 8.05) was used for TIMP-1 structure homology modeling [46]. Protein Homology/analogY Recognition Engine (Phyre; version 2.0) was used for CD82-LEL structure prediction [47]. We used ZDOCK (version 3.0.2) for protein docking [48]. Swiss-PdbViewer was used in automated mode for model structure visualization, analysis, and modification according to the online tutorial [49].

### Chemical cross-linking biotin label transfer

Chemical cross-linking assays were performed as described previously [50] with the following variations. Recombinant TIMP-1 (4 μg) or CD82-LEL (4 μg) was conjugated to Sulfo-SBED at a molar ratio of 1:4 for 30 minutes at room temperature in the dark. Non-reacted cross-linker was removed by dialyzing the reaction mixture in PBS overnight at 4°C. TIMP-1–SBED or CD82-LEL–SBED was incubated with recombinant CD82-LEL (4 μg) or TIMP-1 (4 μg), respectively, for 30 minutes at room temperature. For self–cross-linking, TIMP-1–SBED or CD82-LEL–SBED was incubated alone for 30 minutes at room temperature. The final reaction volume was 100 μL. Cross-linking was initiated by exposure to a UV lamp (365 nm, 8 × 5 watt bulbs, 5 cm lamp–sample distance) on ice for 20 minutes. The sample was split into two new microcentrifuge tubes containing 16 μL each. To one tube, we added 1/5 volume of reducing sample buffer (final DTT concentration, 100 mM). To the other tube, we added 1/5 volume of non-reducing sample buffer. The tubes were incubated for 30 minutes at room temperature, subjected to SDS-PAGE, and immunoblotted with streptavidin–horseradish peroxidase conjugate.

### Competitive binding analysis

Recombinant CD82-LEL was radioiodinated to 1200 Ci/mmol radioactivity. Recombinant TIMP-1 (10 ng, in duplicate) was incubated with an equal amount of 125I–CD82-LEL in the absence or presence of 15- and 225-fold excess unlabeled competitor CD82-LEL for 30 minutes at room temperature in a final volume of 200 μL. After incubation, TIMP-1 antibody (0.2 mg/mL) and A/G beads were supplemented at 4°C overnight. The beads were centrifuged at 2500 rpm for 5 minutes and washed four times with PBS to remove unbound 125I–CD82-LEL. Lastly, the tubes containing the beads were counted in a γ-counter.

### Force spectroscopy

The force spectroscopy experiment was conducted using a commercial atomic force microscope (NanoWizard II, JPK, Germany). Briefly, TIMP-1 was covalently anchored to a glass coverslip via the amino groups. CD63-LEL or CD82-LEL was linked to the cantilever tip through a polyethylene glycol linker via the thiol group of the cysteine residue. The tip was moved at a speed of 400 nm/s with the z range set as 600 nm. The spring constant of the cantilever was approximately 40 pN/nm.

### Cell transfection

Cells were plated on 6-well plates to achieve 50–60% confluence for plasmid transfection or 30–40% confluence for siRNA transfection by Lipofectamine 2000, according to the manufacturer's protocol. 293A cells were transfected with the indicated plasmids; PANC-1 and MCF-7 were transfected with the indicated small interfering negative control (siNC), siRNAs, or plasmids. After 4-6 h, the growth medium was replaced, and clear cell culture chamber inserts (Corning, USA) were incubated with the cells.

### Plasmid construction and siRNA

The enhanced green fluorescent protein (eGFP)-fused TIMP-1 (TIMP-1–eGFP) expressing plasmid was a gift from Professor Thorgeirsson's Laboratory in Maryland (USA). For pCD82-eYFP construction, the CD82 open reading frame (ORF) was obtained by PCR under standard conditions. The primers produce two amplified products: one product with a *Kpn*I restriction site at the 5’ end (Forward: 5’-ggggtaccatgggctcagcctgtatcaaagtc-3’) and the other product with a *Sma*I restriction site at the 3’ end (Reverse: 5’-tcccccgggtcagtacttggggaccttgctgtag-3’) with the stop codon. The amplified products were then digested with *Kpn*I/*Sma*I and cloned into the eYFP-C1 vector conserving the reading frames of both CD82 and eYFP. The final plasmid was sent to BGI, Inc. (Shanghai, China) for sequencing. To silence cell membrane CD82 expression, siRNAs were synthesized by RiboBio Co. (Guangzhou, China). siRNA sequences are 1) si-h-CD82_001: 5’-GAAGAGGACAACAGCCUUU dTdT-3’; 2) si-h-CD82_002: 5’-GGGCAGUCACUAUGCUCAU dTdT-3’; 3) si-h-CD82_003: 5’- GCAUCGUGACUGAGCUCAU dTdT-3’. Cells were transfected with siRNAs targeting CD82 or siNC for 48 hours, and then harvested for RNA analysis, western blotting, or other related experiments.

### Live cell imaging

PANC-1 cells were plated in 3.5-cm dishes (cellview™, Greiner, Germany) and or. To detecting effect of TIMP-1 protein on translocation of CD82-eYFP fusion protein, PANC-1 was transfected with pCD82-eYFP for 24h. Before imaging, cells went through serum starvation for 4 h. Cultures were maintained at 37°C, 5% CO_2_ during imaging in a heated, gas-perfused chamber (Toka engineering, Japan), and visualized with a laser scanning microscope (f1200, Olympus, Japan). After 30 minutes culture to establish a baseline, recombinant TIMP-1 (100 ng/mL) or TIMP-2 (100 ng/mL) was added to the cultures and cells were imaged for a further 30 minutes. Four frames per minute during imaging. Live cell imaging results were quantified by Imaris8.0. To observe merged area of lysosomes and eGFP-tagged TIMP-1, PANC-1 and MCF-7 were treated with 293A culture supernatant containing eGFP-tagged TIMP-1 for 24h. Before imaging, cells were pretreated with cultures containing Lyso-Tracker Red (Beyotime Biotechnology, dilution: 1:20000) for 2 h.

### Wound closure assay

Cells were plated in 3.5-cm dishes and transfected with the indicated siRNAs or siNC for 24 h. Cells were divided into 12-well plates to achieve 70-80% confluence and then wounded by dragging a plastic pipette tip across the monolayer surface three times, followed by gentle washing with PBS twice. Cells were treated with culture medium containing 5% fetal bovine serum plus the indicated concentrations of TIMP-1, cyclopamine, TIMP-2, or DN–TIMP-1. Images of the wounds were recorded with a Leica DM IRB inverted microscope (Solms, Germany).

### Flow cytometry

Cells were harvested, washed three times with pre-chilled PBS and fixed with 1 ml 75% ice-cold ethanol at −20°C overnight. Cells were washed three times in PBS and resuspended in 500 μl propidium iodide (PI) solution (50 μg/ml in PBS). The solution was incubated in the dark at room temperature for 30 minutes and analyzed by a FACSCalibur flow cytometer using Cellquest Pro software (Becton Dickinson Immunocytometry Systems, BD, USA).

### ATP content

PANC-1 cells were plated in 3.5-cm dishes and transfected with siNC or siRNA for 48 h. Following serum starvation for 6 h, cells were treated with recombinant TIMP-1 (100 ng/mL) for 5, 10, 30 minutes or not treated at 37°C. Cells were lysated with ATP lysis buffer according to the manufacturer's instructions (Beyotime Biotechnology). Cell lysates were centrifuged at 12 000 rpm for 5 minutes at 4°C to collect the supernatant. Luminometer TD–20/20 (TURNOR DESIGHS, USA) was applied for ATP content detection. All data were normalized to the protein concentration in the supernatant.

### Immunohistochemistry

Pancreatic (n=32) and colorectal (n=2) tissue sections were treated with 0.3% hydrogen peroxidase for 5 minutes, followed by 30 minutes blocking with 5% normal goat serum in PBS at room temperature. Primary antibodies (TIMP-1, dilution 1:100; CD82, dilution 1:100) were applied and incubated overnight at 4°C. The sections were then incubated for 30 minutes at 37°C with HRP-labeled goat anti-mouse IgG antibody (1:2 000). The positive signals were visualized by development in DAB solution. Images were recorded with a microscope (Axio scope A1, Zeiss, USA).

### Statistical analyses

Statistically significant differences were determined by unpaired, two-tailed, Student's *t*-test. *P*-values < 0.05 were considered statistically significant. Analysis of variance was used to calculate differences between groups where appropriate. Receiver operating characteristics curves were generated on the basis of the comparison between lymph node metastasis group and primary tumor group.

## SUPPLEMENTARY MATERIALS FIGURES AND TABLES




