# Supplementary material for: TIMP-1 and CD82, a promising combined evaluation marker for PDAC
Source: Oncotarget. 2016 Dec 24;8(4):6496–512. doi: 10.18632/oncotarget.14133 (PMC5351648; doi:10.18632/oncotarget.14133)
Supplement: Supplementary file 1 [file oncotarget-08-6496-s001.pdf]

TIMP-1 and CD82, a promising combined evaluation marker for PDAC

SUPPLEMENTARY FIGURES AND MOVIE

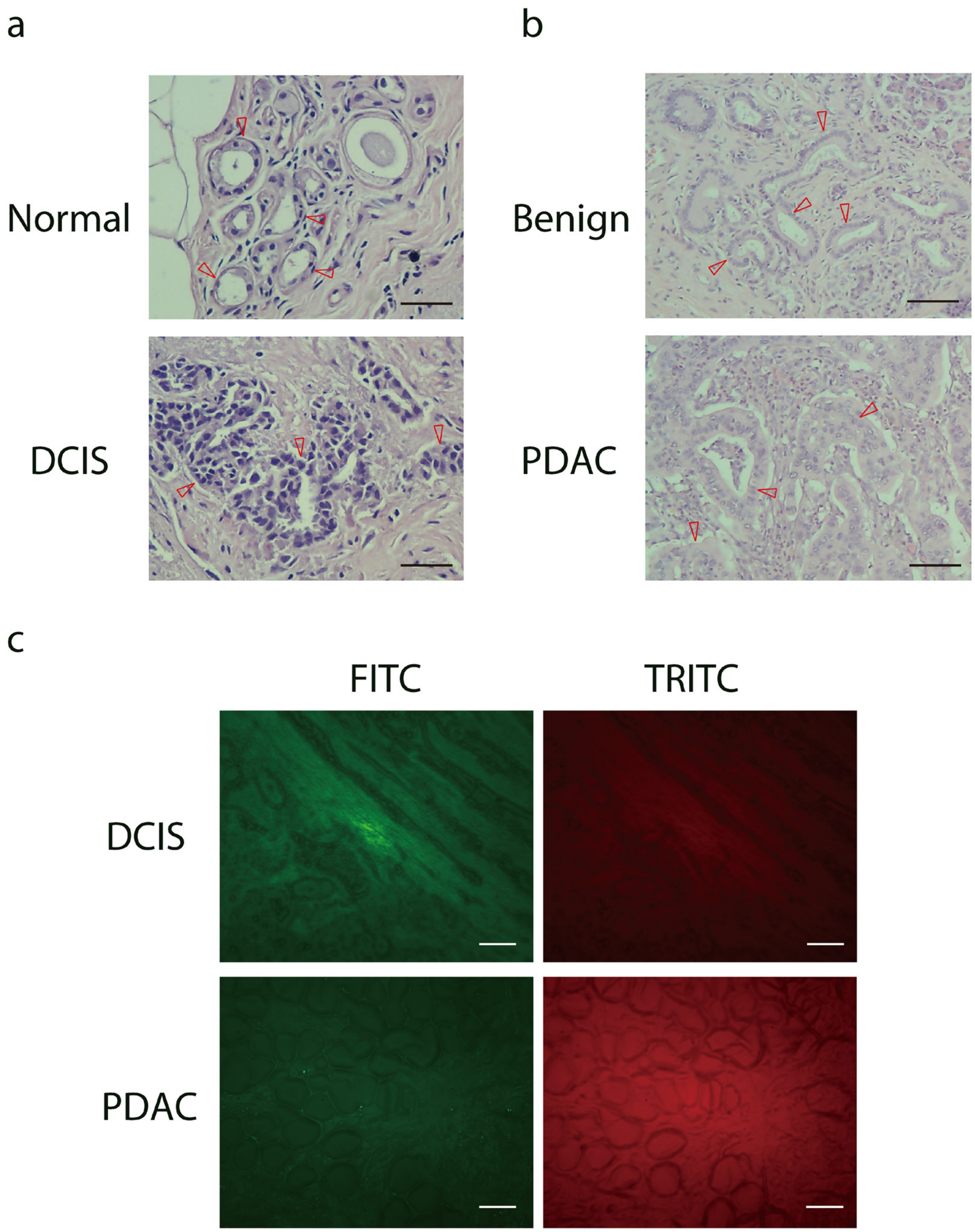

Supplementary Figure 1: HE staining and negative controls of DCIS and PDAC biopsies in Figure 2. a. HE staining of DCIS in Figure 2b, scale bar = 200μm. **b.** HE staining of PDAC in Figure 2c, scale bar = 200μm. **c.** negative controls of Figure 2b and 2c (only secondary Ab used), scale bar = 200μm.

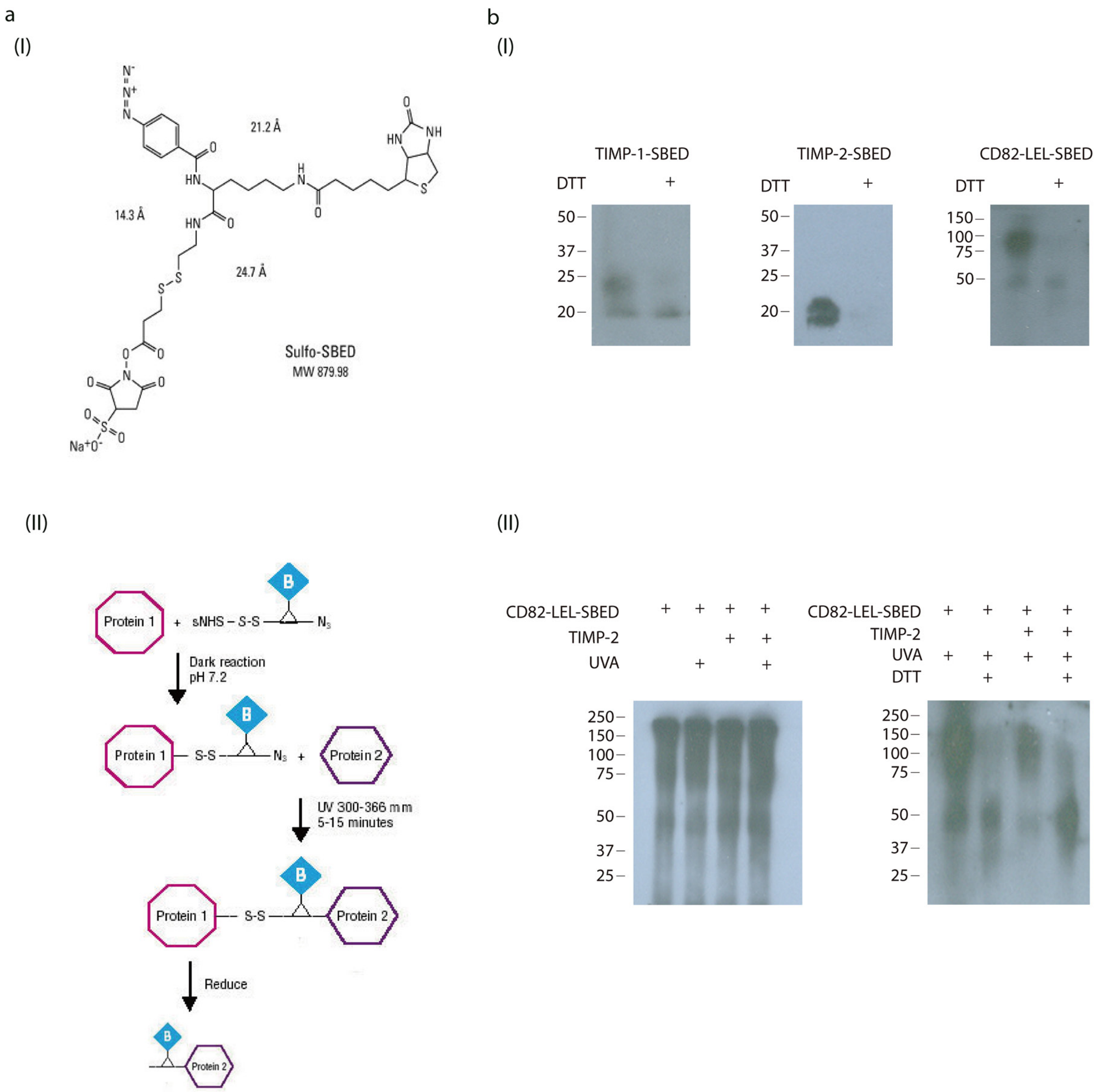

Supplementary Figure 2: TIMP-2 and CD82-LEL binding by chemical cross-linking experiments. a. (I) structural and functional properties of Sulfo-SBED (Thermo Scientific™) for biotin label transfer. (II) experimental strategy for Sulfo-SBED biotin label transfer and analysis by western blotting. **b.** (I) recombinant TIMP-1, TIMP-2 and CD82-LEL-SBED treated with DTT. (II) cross-linking experiments between CD82-LEL-SBED and TIMP-2.

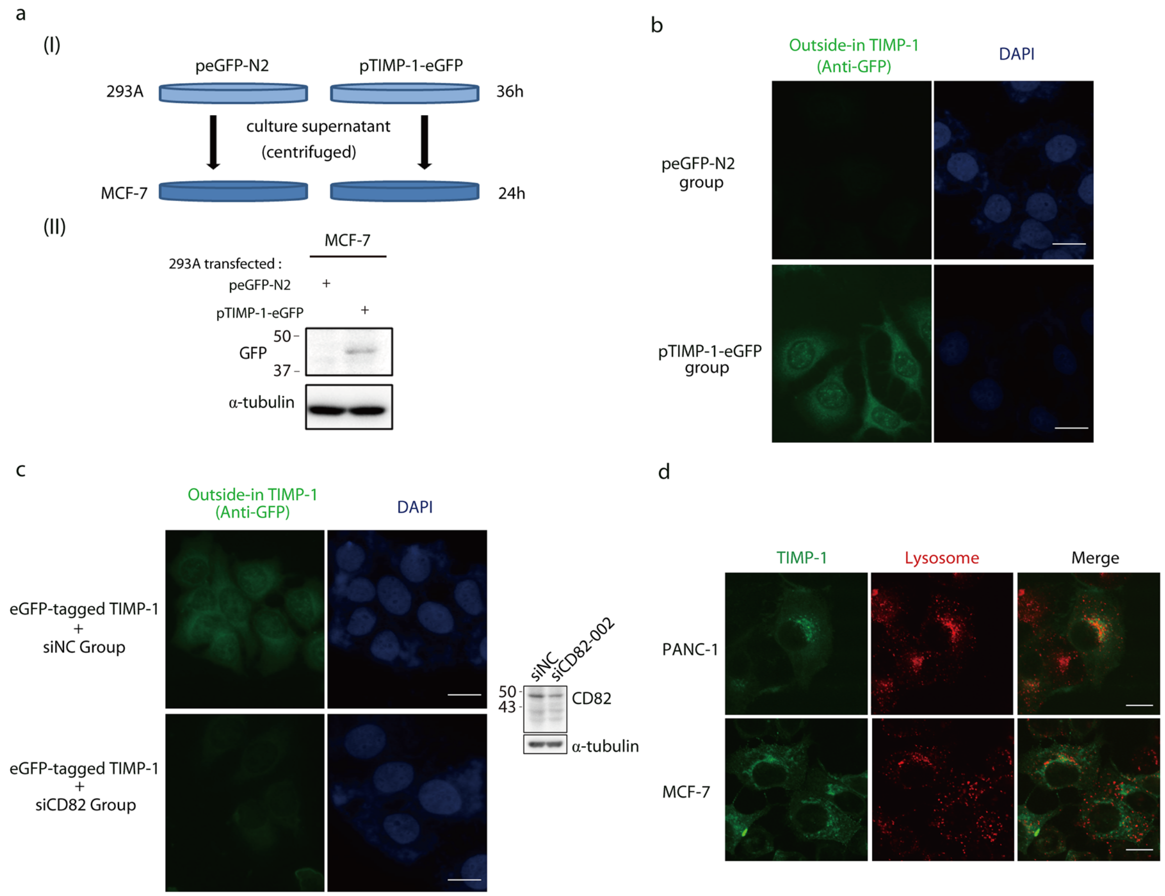

Supplementary Figure 3: TIMP-1 membrane–cytoplasm translocation in MCF-7 cells requires CD82. a. (I) Culture system model of 293A cells and MCF-7 cells. (II) MCF-7 cells were treated with culture supernatant from eGFP/eGFP-TIMP-1 transfected 293A cells. Outside-in GFP was detectable in MCF-7 cells. Cell lysates were immunoblotted with anti-GFP and anti–α-tubulin mAbs. **b.** TIMP-1–eGFP membrane–cytoplasm translocation. The process was the same as that described for Figure 4c. Scale bar = 20μm. **c.** CD82 mediated TIMP-1–eGFP membrane–cytoplasm translocation. The process was the same as that described for Figure 4d. Scale bar = 20μm. **d.** lysosomes were labeled with red fluorescence in MCF-7 and PANC-1 cells which have been treated with culture supernatant containning eGFP-tagged TIMP-1before. Pearson's coefficient of the co-localization was 0.79±0.17 in PANC-1, 0.13±0.09 in MCF-7.

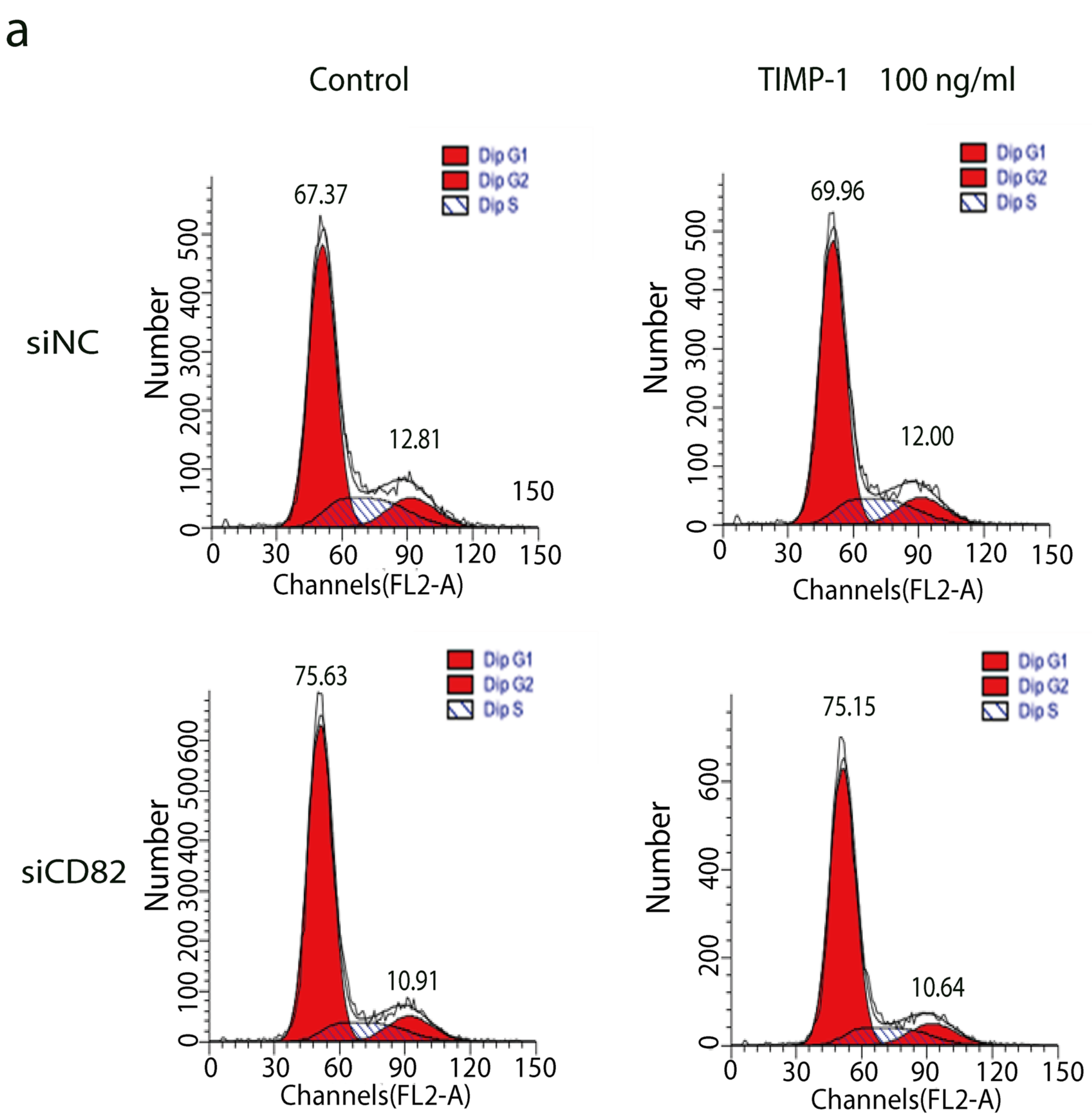

Supplementary Figure 4: TIMP-1 did not affect the cell cycle. a. Cells were transfected with siNC or siCD82 and treated with TIMP-1 for 18 h. Cells were harvested for flow cytometry with PI staining.

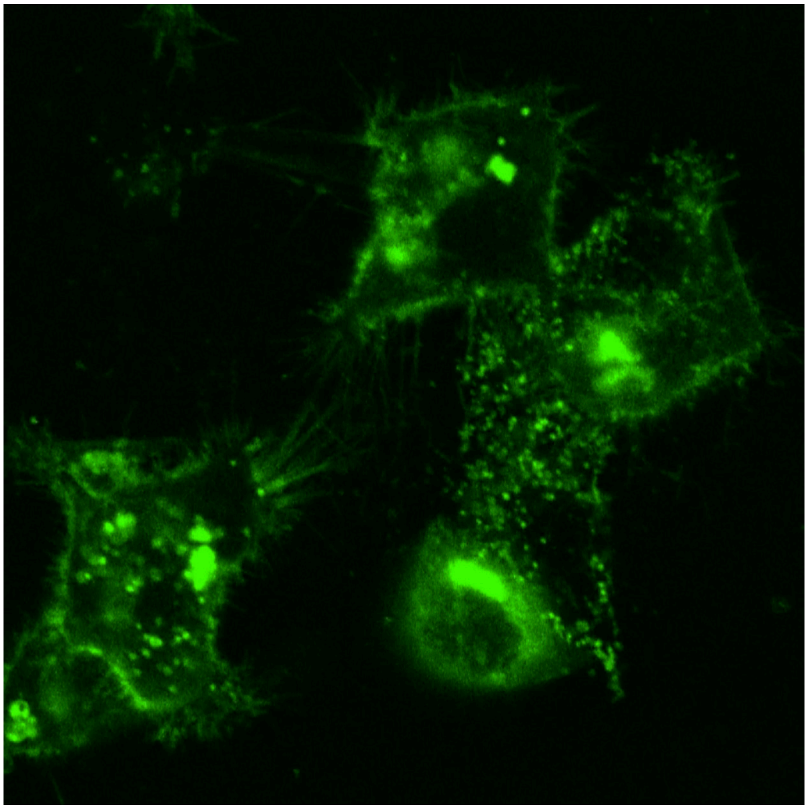

Supplementary Movie 1: CD82-eYFP fusion protein was transferred into cytoplasim as soon as addition of TIMP-1 recombinant protein (100 ng/ml). Filopodia present on the surface of PANC-1 cells underwent retraction within 15 min after TIMP-1 was applied. Four frames per minute were captured during 30-minute imaging

See Supplementary Movie 1
